# Supplementary material for: Using de-identified electronic health records to research mental health supported housing services: A feasibility study
Source: PLoS One. 2020 Aug 20;15(8):e0237664. doi: 10.1371/journal.pone.0237664 (PMC7444482; doi:10.1371/journal.pone.0237664)
Supplement: S1 Table — (DOCX) [file pone.0237664.s001.docx]

| **S1 Table** Original values in structured fields and their grouping term | | |
| --- | --- | --- |
| **Demographics field value**  **‘accommodation_status_desc’** | **CPA field value**  **‘cpa_accommodation_desc’** | **Group term** |
| CH00 Accommodation with other | Accommodation with other (not specialist mental health) care support | Other |
| CH01 Foyer - accommodation for young people aged 16-25 who are homeless or in housing need | Foyer - accommodation for young people aged 16-25 who are homeless or in housing need | Other |
| CH02 Refuge | Refuge | Other |
| CH03 Non-Mental Health Registered Care Home | Non-Mental Health Registered Care Home | Other |
| CH09 Other accommodation with care and support | Other accommodation with care and support (not specialist mental health) | Other |
| CJ00 Accommodation with criminal justice support | Accommodation with criminal justice support | Criminal justice |
| CJ01 Bail/Probation hostel | Bail/Probation hostel | Criminal justice |
| CJ02 Prison | Prison | Criminal justice |
| CJ03 Young Offenders Institute | Young Offenders Institution | Criminal justice |
| CJ09 Other accommodation with criminal justice support such as ex-offender support | Other accommodation with criminal justice support such as ex-offender support | Criminal justice |
| … | Detention Centre | Criminal justice |
| HM00 Homeless | Homeless | Homeless/Temporary |
| HM01 Rough sleeper | Rough sleeper | Homeless/Temporary |
| HM02 Squatting | Squatting | Homeless/Temporary |
| HM03 Night shelter/emergency hostel/Direct access hostel | Night shelter/emergency hostel/Direct access hostel | Homeless/Temporary |
| HM04 Sofa surfing | Sofa surfing | Homeless/Temporary |
| HM05 Placed in temporary accommodation by Local Authority | Placed in temporary accommodation by Local Authority | Homeless/Temporary |
| HM06 Staying with friends/family as a short term guest | Staying with friends/family as a short term guest | Homeless/Temporary |
| HM07 Other homeless | Other homeless | Homeless/Temporary |
| HS00 Acute/long stay healthcare residential facility/hospital | Acute/long stay healthcare residential facility/hospital | Hospital |
| HS01 NHS acute psychiatric ward | NHS acute psychiatric ward | Hospital |
| HS02 Independent hospital/clinic | Independent hospital/clinic | Hospital |
| HS03 Specialist rehabilitation/recovery | Specialist rehabilitation/recovery | Hospital |
| HS04 Secure psychiatric unit | Secure psychiatric unit | Hospital |
| HS05 Other NHS facilities/hospital | Other NHS facilities/hospital | Hospital |
| HS09 Other acute/long stay healthcare residential facility/hospital | Other acute/long stay healthcare residential facility/hospital | Hospital |
| MA00 Mainstream Housing | Mainstream Housing | Mainstream housing |
| MA01 Owner occupier | Owner occupier | Mainstream housing |
| MA02 Settled mainstream housing with family/friends | Settled mainstream housing with family/friends | Mainstream housing |
| MA03 Shared ownership scheme | Shared ownership scheme e.g. Social Homebuy Scheme | Mainstream housing |
| MA04 Tenant - Local Authority/Arms Length Management Organisation/Registered Landlord | Tenant - Local Authority/Arms Length Management Organisation/Registered Landlord | Mainstream housing |
| MA05 Tenant - Housing Association | Tenant - Housing Association | Mainstream housing |
| MA06 Tenant - private landlord | Tenant - private landlord | Mainstream housing |
| MA09 Other mainstream housing | Other mainstream housing | Mainstream housing |
| MH00 Accommodation with mental health care support | Accommodation with mental health care support | Mental health supported |
| MH01 Supported accommodation | Supported accommodation | Mental health supported |
| MH02 Supported lodgings | Supported lodgings | Mental health supported |
| MH03 Supported group home | Supported group home | Mental health supported |
| MH04 Mental Health Registered Care Home | Mental Health Registered Care Home | Mental health supported |
| MH09 Other accommodation with mental health care and support | Other accommodation with mental health care and support | Mental health supported |
| ML00 Mobile accommodation | Mobile accommodation | Other |
| NULL | NOT RECORDED | Not recorded or unknown |
| OC96 Not elsewhere classified | Not elsewhere classified | Not recorded or unknown |
| OC97 Not specified | UNKNOWN | Not recorded or unknown |
| OC98 Not applicable | … | Not recorded or unknown |
| OC99 Not known | Not known | Not recorded or unknown |
| SH00 Sheltered Housing | Sheltered Housing | Not recorded or unknown |
| SH01 Sheltered housing for older persons | Sheltered housing for older persons | Not recorded or unknown |
| SH02 Extra care sheltered housing | Extra care sheltered housing | Not recorded or unknown |
| SH03 Nursing Home for older persons | Nursing Home for older persons | Not recorded or unknown |
| SH09 Other sheltered housing | Other sheltered housing | Not recorded or unknown |
