# Supplementary material for: Using de-identified electronic health records to research mental health supported housing services: A feasibility study
Source: PLoS One. 2020 Aug 20;15(8):e0237664. doi: 10.1371/journal.pone.0237664 (PMC7444482; doi:10.1371/journal.pone.0237664)
Supplement: S3 Table — (DOCX) [file pone.0237664.s003.docx]

| **S3 Table.**Search results for the demographics structured field. | | |
| --- | --- | --- |
| Type of accommodation^1^ | Total number ever recorded^2^ | Total distinct individuals per type of accomm.^3^ |
| Mental health supported | 2179 | 882 |
| Not recorded or unknown | 36577 | 17899 |
| Mainstream housing | 20587 | 9087 |
| Homeless/Temporary | 4101 | 1492 |
| Nursing home / Sheltered housing | 561 | 267 |
| Hospital | 373 | 159 |
| Other | 521 | 225 |
| Criminal justice | 166 | 72 |
| TOTAL | 65065 | 30083 |
| Date range for search results: 01-Jan-2008 to 31-Dec-2017. ^1^There are a total of 50 values for this field in the CIFT Research Database. They have been grouped here with appropriate labels for clarity. For example, the value 'Mental health supported' consists of the following database values: 'MH00 Accommodation with mental health care support', 'MH01 Supported accommodation', 'MH02 Supported lodgings', 'MH03 Supported group home’, ‘MH04 Mental Health Registered Care Home' and 'MH09 Other accommodation with mental health care and support'. See Table S1 for the full list of original values and their grouping term. ^2^Individuals can appear for more than one type and within the same type more than once. ^3^Individuals can appear for more than one type but only once within the same type. | | |
